# Role of noncoding RNA as a pacemaker in cancer stem cell regulation: a review article

**DOI:** 10.1186/s43046-025-00266-2

**Published:** 2025-03-24

**Authors:** Yasmin M. Attia, Samer A. Tadros, Sally A. Fahim, Doaa M. Badr

**Affiliations:** 1 Pharmacology Unit, Cancer Biology Department, National Cancer Institute, Cairo University, Kasr Al Eini Street, Fom El Khalig, Cairo, 11796 Egypt; 2 Department of Biochemistry, Faculty of Pharmacy, 110123october University for Modern Sciences and Arts (MSA), 6th of October City, Egypt; 3 Department of Biochemistry, School of Pharmacy, Newgiza University (NGU), Newgiza, Km 22 Cairo-Alexandria Desert Road, Giza, 12577 Egypt

**Keywords:** Cancer, Cancer stem cells, Non-coding RNA, MicroRNA, Long non-coding RNA

## Abstract

Accumulated evidence supported the crucial role of a tiny population of cells within the tumor called cancer stem cells (CSCs) in cancer origination, and proliferation. Additionally, these cells are distinguished by their self-renewal, differentiation, and therapeutic resistance capabilities. Interestingly, many studies recorded dysregulation of different types of noncoding RNAs, such as microRNA (miRNA) and long non-coding RNA (LncRNA), in cancer cells as well as CSCs. Moreover, several studies also supported the regulation of the transcription factors and signaling pathways required for CSC progression by these noncoding RNAs. However, the exact biological functions of all these noncoding RNAs are not well understood yet. These findings are of great interest, implying usage of noncoding RNA as therapeutic tool to target these cells. In this review, we provide an insight into how noncoding RNAs regulate CSCs and how this correlation is manipulated to develop new therapies to eradicate cancer cells successfully.

## Background

Cancer remains the main cause of death all over the world [31], as many long-established cancer therapies, surgical intervention, chemotherapy, and radiotherapy showed a high rate of failure and some patients suffer from the relapse after the initial responses [123]. One of the recent theories that explain such relapse is that all tumors are composed of cells that exhibit diversity in both appearance and genetic makeup. Within this diverse population of cancer cells lies a subset with characteristics resembling stem cells, including the abilities of self-renewal and multipotency, referred to as cancer stem cells (CSCs) or tumor-initiating cells. These cells possess the capacity to propel tumor progression and differentiate into various cell types, contributing to the diversity observed within the tumor itself [25]. The molecular signaling pathways responsible for controlling CSCs are disrupted, leading to irregularities that impact the self-renewal, proliferation, survival, and differentiation of CSCs. These signaling pathways include JAK/STAT, Wnt, NF-κB, and PI3K/Akt/mTOR signaling pathways [139].

It is approximated that around 90% of the genome is actively transcribed into RNAs. Yet only 1.5–2.0% of the human genome is thought to comprise protein-coding genes. Several classes of ncRNA have been recognized within the last two decades. The size of ncRNA varies from 20 nucleotides to several hundred nucleotides. According to their transcript sizes, ncRNA could be categorized into two major categories: small ncRNAs less than 200 bp, such as Piwi-associated RNAs (piRNAs), microRNAs (miRNAs), and small nucleolar RNAs (snoRNAs), and long ncRNAs (lncRNAs) greater than 200 bp [50]. Increasing research has detailed the possible roles of lncRNAs in CSCs due to their participation in oncogenic and tumor-suppressive pathways [20, 90].

The aim of our review is exploring the functions of ncRNAs within the realm of CSCs in different types of cancer and discussing their potential therapeutic uses.

## Discovery of cancer stem cells

Cancer remains the main cause of death all over the world. Despite the enormous effort and research to understand cancer molecular biology and the great improvements in cancer diagnosis and therapies, the number of deaths from cancer is still rising every year [31], as many long-established cancer therapies, surgical intervention, chemotherapy, and radiotherapy showed a high rate of failure and some patients suffer from the relapse after the initial responses [123]. Therefore, many theories have been revealed to explain this drawback, and many scientists tried to discover new molecules to counteract this relapse.

One of the recent theories that explains such relapse was discovered in 1997 when two scientists succeeded in the extraction of distinctive leukemia cells that had a surface marker CD34^+^, but not CD38^−^, and these cells are able to originate tumors in NOD/SCID mice that were analogous to the giver [18]. These subpopulations of cancer cells were termed CSCs or stem-like cancer cells. The solid tumor CSCs were recognized at the beginning in breast cancer (CD44^+^CD24^−/low^Lin^−^) [1], then they were isolated from many other types as colorectal cancer [29] and brain cancer [56].

### Cancer stem cells vs normal stem cells

CSCs have the same characteristics of normal stem cells (NSCs) showing the capability of self-renewal and differentiation. It was hypothesized that targeting these cells which represent the main origin of cancer could be a hopeful approach for cancer cure as well as preventing the relapse of cancer.

Since NSCs and CSCs share many features as mentioned above, it is advisable to study NSCs first to understand the nature of CSCs. Some of these features include the ability of NSCs to divide asymmetrically, producing a new cell with the ability to self-renew and amplify stem cell self-renewal, as well as a transit amplifying cell known as a progenitor cell. These new stem cells have an important feature demonstrated by an ability to maintain the undifferentiated stem cell population throughout an individual’s lifetime [48]. Moreover, stem cells can be stimulated and proliferate due to local or systemic signals, and any disturbance in the asymmetric division will lead to an increase in the number of stem cells and developing cancer eventually [48]. Stem cells have the ability to differentiate into tissue-specialized cells. These differentiated cells will either die or will be substituted by different new ones, while the stem cells remain constant over a lifetime. There are two categories of NSCs: embryonic stem (ES) and adult stem (AS) cells [99]. ESCs are located within the inner cell mass of the blastocyst, which is a hollow sphere of cells that develops approximately 3 to 5 days after the fertilization of the egg by sperm in humans. This type of stem cells is *pluripotent*; in other words, these cells can develop to any cell type found in the mature body, with the exception of cells forming the placenta and umbilical cord. These cells offer a precious tool for investigating the regular development and unusual illness besides exploring countless drugs and new compounds. On the other hand, AS cells, or the so-called tissue specific cells cells, yield a limited group of specialized cells distinctive for a certain tissue. This feature makes AS cells more specified than ES cells. Based on their ability to produce many types of cells, they act as a pool of long-lived cells, which is very important in hemostatic regulation of adult mature tissues that go through nonstop turnover. Induced pluripotent stem (iPS) cells are another category of stem cells that have been developed by switching tissue-specific cells to cells that act like ESCs. iPS cells are considered a significant method to study the normal development and disease progression and test new therapies [99].

### Origin of CSCs

**Fig. 1:**
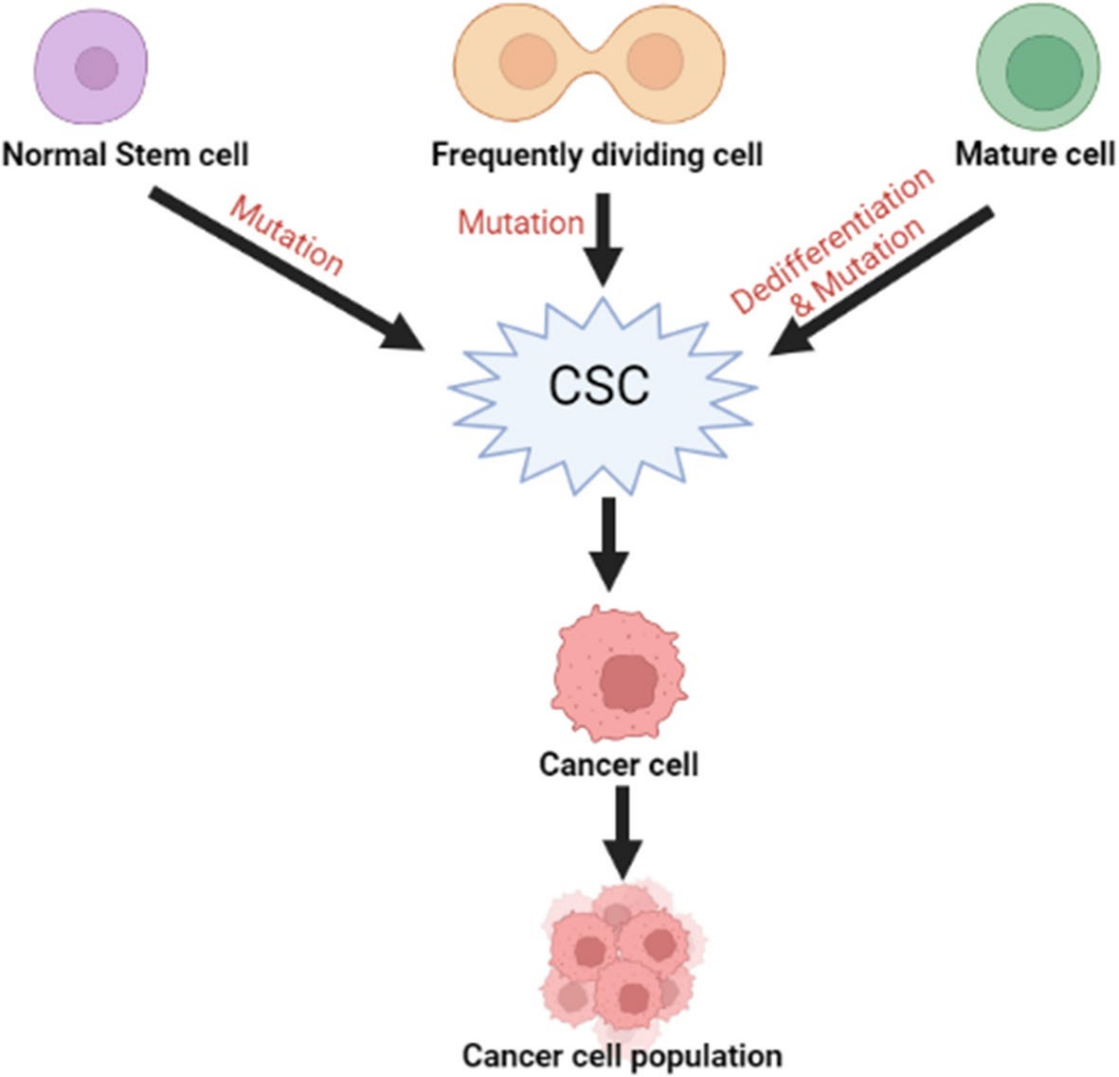
Schematic representation of the origin of CSCs. CSC, cancer stem cells

The initial theory proposed that CSCs originate from pre-existing stem cells. Then these NSCs undergo many mutations required for tumor formation and metastasis and become CSCs [109]. The second theory suggested that CSCs originate from progenitor or precursor cells which are semi-differentiated cells that exist in fetal and adult tissues and frequently divide to produce more mature cells [79]. This phenomenon is coupled with tissues that have a high rate of cell turnover such as the skin or gut. Genetic mutation buildup is the main factor that stimulates the tumor initiation [94]. A third theory suggested that CSCs may arise from mature, differentiated cells that undergo a process of de-differentiation, acquiring stem-like characteristics in some manner (Fig. 1). It is assumed that multiple genetic alterations are implied in the de-differentiation of cells to form stem-like cells. Therefore, a large number of cells in the tissue might have tumorigenic potential, and in fact, some of them would stimulate the tumor initiation eventually [136].

### Characteristics of CSCs

CSCs have high stemness characters resulting from the high expression level of stem cell genes, a mutual feature with either ESCs and adult stem cells, in addition to their ability to grow as spheres [73]. CSCs are not eradicated by conventional chemotherapies or radiotherapy due to the high expression of anti-apoptotic proteins [104] or the multidrug resistance transporter, ABC [153]. CSCs are the main reason behind the resistance of these cancers to the traditional chemotherapies. Additionally, it was found that CSCs have one or more abnormalities in many signaling pathways as Notch, Hedgehog (HH), and Wnt [116]. Numerous researchers have made efforts to eliminate CSCs by targeting CSC-specific markers, cellular signaling pathways associated with CSCs, and the microenvironment supporting CSCs. Various studies have explored strategies such as targeting CD133, a known CSC marker, through drug binding to CD133 antibodies or CD133 aptamer-conjugated nanoparticles. However, these approaches have shown limited effectiveness in achieving significant anti-CSC effects [116]. Despite various research, most of these studies showed limitations of CSC targeting strategies as CSCs exist as heterogeneous population and a single CSC marker does not suitably differentiate between CSCs and non-CSCs [103], so CSC marker-negative or differentiation marker-positive cancer cells might both stimulate the tumor formation [58]. Likewise, activation of CSC-specific signaling pathways might possibly differ within a tumor, so the inhibition of a single pathway cannot significantly influence the entire CSCs [94].

### CSC signaling pathways

**Fig. 2:**
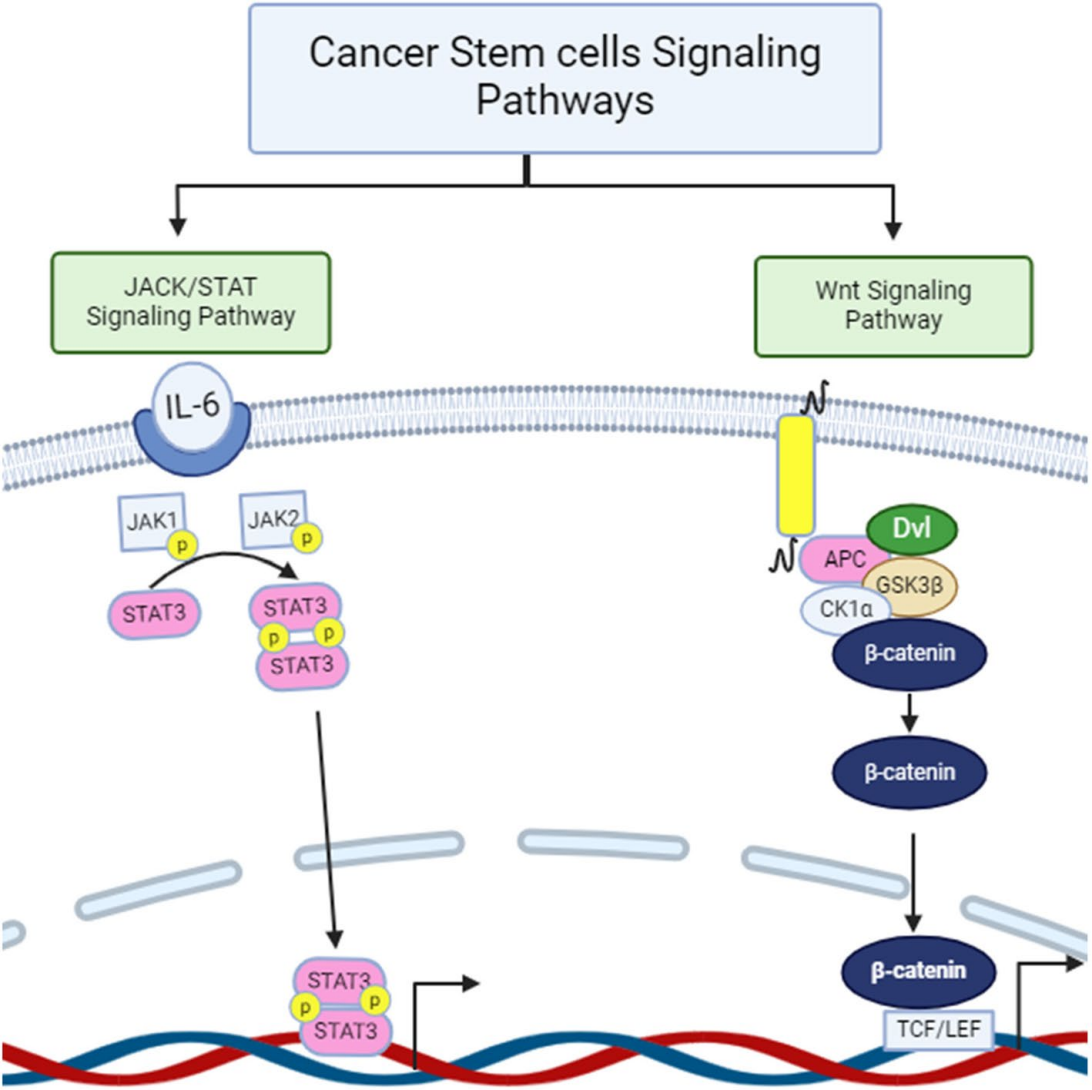
JAK-STAT and Wnt signaling pathways in cancer stem cells. JAK, Janus kinase; STAT, signal transducer and activator of transcription; Wnt, Wingless-related integration site; APC, Adenomatous polyposis coli; Dvl, Dishevelled

**Fig. 3:**
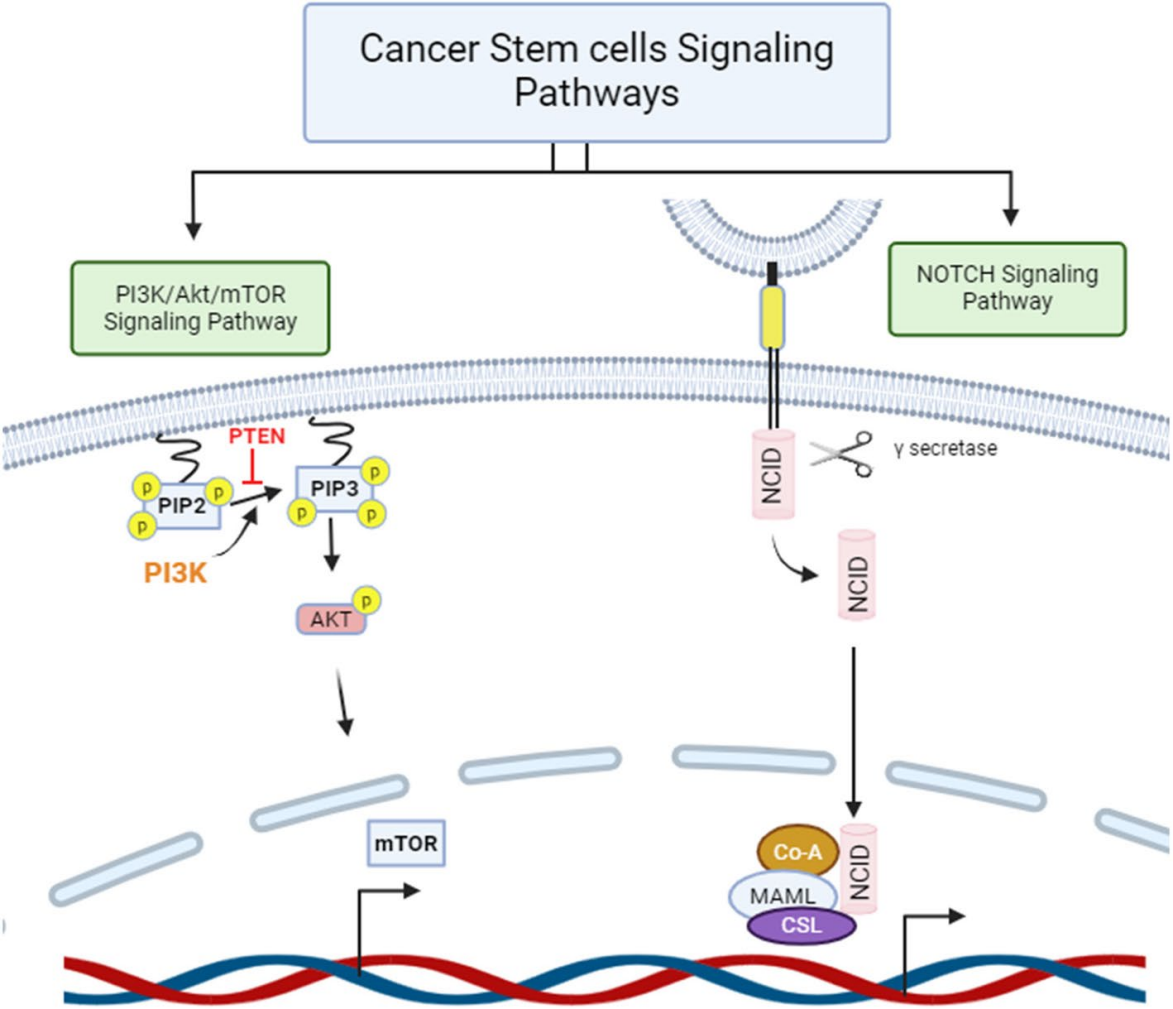
PI3K/AKT/mTOR and NOTCH signaling pathways in cancer stem cells. AKT, protein kinase B; mTOR, mammalian target of rapamycin; PI3K, phosphatidylinositol 3-kinase; PIP2, phosphatidylinositol 4,5-bisphosphate; PIP3, phosphatidylinositol (3,4,5)-trisphosphate; PTEN, phosphatase and tensin homolog; Co-A, coactivator; CSL, CBF1 Suppressor of Hairless Lag-1; MAML, mastermind; NICD, Notch intracellular domain

The molecular signaling pathways responsible for controlling CSCs are disrupted, leading to irregularities that impact the self-renewal, proliferation, survival, and differentiation of CSCs. Therefore, various molecular approaches, such as examining the expression of cell surface proteins (like CD44 and CD133), intracellular markers (such as aldehyde dehydrogenase, ALDH), stemness-related genes (including OCT4 and SOX2), and conducting phenotypic tests (like tumor sphere formation and serial in vivo tumor transplantation), are employed to assess stemness [27, 120]. These signaling pathways include JAK/STAT, Wnt, NF-κB, and PI3K/Akt/mTOR illustrated in Figs. 2 and 3.

#### JAK/STAT signaling pathway

The activation of the JAK/STAT signaling pathway occurs when various ligands, such as interleukins and interferons, bind to their specific receptors. This binding event triggers receptor oligomerization and the clustering of JAK proteins (JAK1-3 and TYK2), leading to the phosphorylation and activation of the JAK proteins. Upon binding with JAKs, STATs undergo phosphorylation, resulting in dimerization and penetration of the nuclear membrane, initiating the transcription of target genes [101].

The JAK/STAT pathway plays a role in maintaining self-renewal characteristics in embryonic stem cells, hematopoiesis, and neurogenesis. Stem-like cells extracted from prostate cancer cells were found to exhibit elevated expression of several genes associated with JAK/STAT signaling, including IFNK, IFNGR, IL6, CSF2, and STAT1 [154]. Blocking STAT3 through chemical inhibition in CSCs hinders proliferation and tumor sphere formation while reducing the expression of stem cell genes in the neurons [34, 122]. JAK/STAT signaling has been linked to CSC-driven metastasis, as evidenced by colon CSCs isolated from stage IV clinical tumor samples [102]. Examination of CSCs from individuals with leukemia revealed continuous activation of JAK/STAT signaling. Treatment with a JAK1/2 inhibitor led to decreased CSCs’ survival [42].

#### Wnt signaling pathway

Wnt signaling pathway is also involved in CSCs. The Wnt signaling pathway comprises three separate cascades: (1) the canonical pathway (including β-catenin, TCF, and LEF; (2) the non-canonical pathway that operates independently of β-catenin; and (3) the non-canonical Wnt-calcium pathway. The canonical Wnt pathway is linked to the expansion and maintenance of stem cells and participates in the proliferation and differentiation of progenitor cells [146]. SAM68 functions as a new transcriptional regulator that specifically targets CSCs through the Wnt/β-catenin pathway [14]. Moreover, Niclosamide, an anthelmintic drug, was recognized as a suppressor of the Wnt/β-catenin pathway and demonstrated anti-cancer characteristics by specifically aiming at ovarian CSCs [83].

#### Notch signaling pathway

The Notch receptor family consists of four members (Notch 1–4) that have the ability to interact with five different ligands. The expression of NOTCH can be either increased or reduced. The key cancer stem cell markers associated with the Notch pathway include CD133, Musashi-1, CD44, EpCAM, CD166, and Bmi1 [110]. Elevated Notch 1, 2, and 3 levels wer observed in brain, pancreatic tumors, and breast cancer, while decreased expression is common in colorectal cancer. In breast cancer, the roles of NOTCH genes vary depending on the molecular subtype of the tumor [118]. For instance, NOTCH1 can trigger HER2 transcription, leading to an escalation in mammary and breast CSCs. As the Notch pathway plays a role in stem cell specialization, it can facilitate the formation of ER + luminal tumors. The increased expression of Notch2 and Notch3 contributes to the transformation of progenitor cells into luminal-type cells, with Notch3 enhancing invasion. Conversely, Notch4 is predominant in the breast CSC group and is found in basal cells. Notch signaling stimulates the activation of genes necessary for epithelial-to-mesenchymal transition (EMT), a critical process in CSCs. EMT is an inherent mechanism crucial for the metastatic dissemination of tumors. Studies suggest that Notch1 activation suppresses E-cadherin, leading to EMT in breast cancer cells. Moreover, Notch activation under hypoxic conditions results in the reduction of E-cadherin and β-catenin levels, enhancing the invasion of breast cancer cells [13].

#### PI3K/Akt/mTOR signaling pathway

The PI3K signaling pathway is commonly overactive in various cancers, playing a crucial role in CSCs by regulating tumor development and stemness [69, 125]. PTEN is a signaling molecule and a significant inhibitor of tumor growth. Genetic alteration in PTEN led to resistance to apoptosis and increased cell migration [89]. The inhibition of this pathway alleviates chemoresistance and stemness in ovarian cancer and lung cancer cells [32, 59] and is considered a potential therapeutic target in glioblastoma [4].

### Non-coding RNA (ncRNA)

In 1958, Francis Crick made a significant discovery known as the central dogma, where DNA eventually leads to protein synthesis [28]. It was widely believed that proteins served as the primary functional end products of genetic information, with less than 2% of the genome dedicated to encoding these proteins. However, recent evidence has emerged suggesting that a substantial portion of mammalian and other complex organism genomes is transcribed into non-coding RNAs (ncRNAs), many of which undergo splicing and are further processed into smaller fragments.

The groundbreaking discovery of the first small RNAs, namely lineage defective 4 (lin-4) [76] and lethal 7 (let-7) [108], in the nematode *Caenorhabditis elegans* revolutionized the field of genetics. These studies revealed that certain RNAs, called ncRNAs, are devoid of protein-coding regions and possess functional roles critical for growth and development. Several classes of ncRNA have been recognized within the last two decades. The size of ncRNA varies from 20 nucleotides to several hundred nucleotides. According to their transcript sizes, ncRNA could be categorized into two major categories: small ncRNAs less than 200 bp, such as Piwi-associated RNAs (piRNAs), microRNAs (miRNAs), and small nucleolar RNAs (snoRNAs), and long ncRNAs (lncRNAs) greater than 200 bp [50].

#### Correlation between miRNA and CSCs

**Fig. 4:**
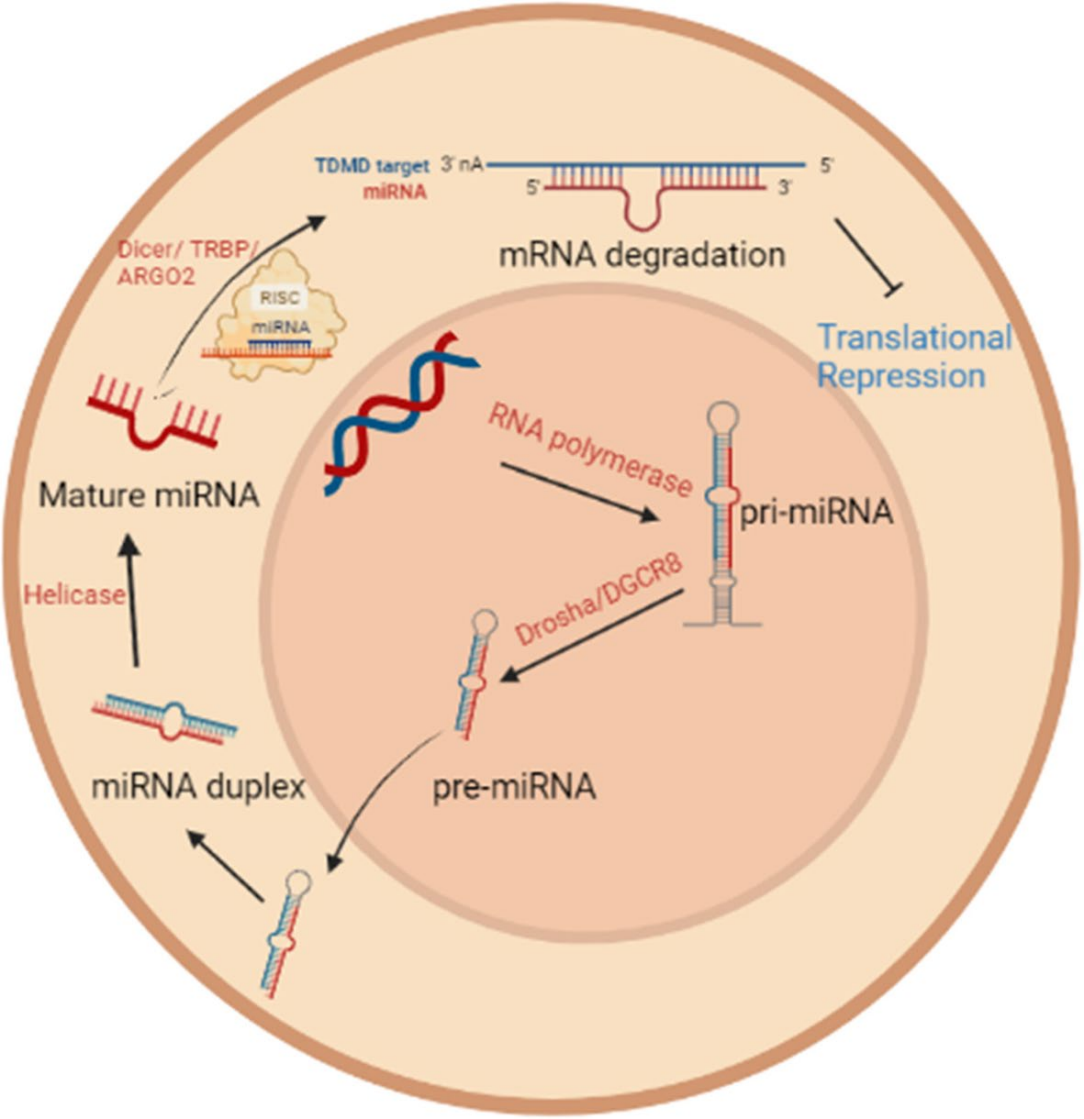
Visual representation of the miRNA biogenesis process. miRNA, microRNA; Pri-miRNA, primary miRNA; RISC, RNA-induced silencing complex; TRBP, transactivation response RNA binding protein

MiRNAs, which are 20–25 nucleotides long, act by regulating the translation process by base pairing to the 3`end of the mRNA [54]. The biogenesis of miRNAs initiates in the nucleus through transcription by RNA polymerase II, resulting in the formation of long primary hairpin miRNAs (pri-miRNAs) that are further cleaved by the endonuclease Drosha [66, 142]. Subsequently, the cleaved hairpin intermediate (~ 70 nucleotides), known as pre-miRNA, is transported to the cytoplasm. The pre-miRNAs undergo further cleavage by the endonuclease, dicer-1. This cleavage generates a short double-stranded duplex that helicase enzyme unwound into a mature miRNA [22, 107] (Fig. 4).

It was found that miRNA aberrant expression is correlated to tumor progression [93]. In 2006, it was the first time for scientists to discover that miR-15 and miR-16 were missing in chronic lymphocytic leukemia (CLL) patients [130]. Then after that, multiple miRNAs were noticed to be dysregulated in several classes of cancer [115] as shown in Table 1.

**Table 1:** List of miRNA names and categories as suppressors or activators of cancer progression in each cancer type

Cancer type	MiRNA as suppressors	MiRNA as activators
Breast	Let-7 [30], miR-30c [40], miR-140-5p [48], miR-141 [53]), miR-199a-5p [117], miR-200c [88], miR-205# [106], miR-600 [39]	miR-49 [51], miR-205# [105]
Lung	Let-7b [143]	
Ovarian	Let-7 [6]	miR-134-3p [148]
Prostate	miR-34a [21], miR-141 [86], miR-145 [23]	miR-302/miR-367 [132]
Colorectal	miR-34 [145], tRF-miR-1280 [98]	miR-21 [70], miR-27a [11], miR-199a/b [24]
Brain	miR-34a [64], miR-136-3p [128]	miR-9/9 [77], miR-199b [45]
Pancreatic	miR-200b-3p [129]	miR-181 [5]
Liver	miR-589 [134]	miR-130b [70]
Bladder	miR-139 [133]	
Skin	miR-130a [62]	miR-142-5p [63]
Renal	miR-145 [23]	
Leukemia		miR-99 [144]
Head and neck	miR-15a, miR-126, and miR-16a [67]	miR-196a and miR-196b [2]

**Fig. 5:**
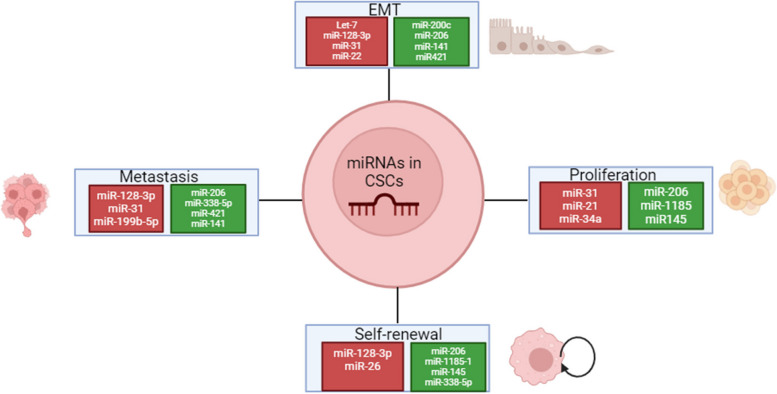
Correlation between miRNAs and the hallmarks of cancer stem cells. miRNA that acts as promotor and suppressor are presented in green and red, respectively. EMT, epithelia mesenchymal transition

Many researchers believe that miRNAs are essential factors in stem cell function, as they have observed differential expression of miRNAs in stem cells compared to other normal tissues [114]. The study revealed that the absence of Dicer-1 (dcr-1), a key player in the processing of precursor miRNAs, in mouse models led to the premature death of the animals during development and a decrease in stem cells within their embryos. This finding underscores the importance of miRNAs in supporting the maintenance of stem cells [15]. Evidence suggests that miRNA could act as a suppressor or activator in pathways that regulate CSC carcinogenesis, metastasis, differentiation, and EMT. Many miRNAs, such as let-7 family, miR-21, miR-26a, and miR-34a, were ascertained as essential regulators in CSCs progression. Let-7 family has a critical function in cancer development and progression through targeting many pathways. Let-7a, b, and c members of let-7 family are negative regulators of EMT and the RNA binding protein, Lin28B in pancreatic and prostate cancer cells [81]. Moreover, it was found that the expression of let-7 family was low in CSCs, in comparison to its parental cells [47]. Interestingly, let-7 decreased breast cancer cell division and mammosphere formation in mouse xenograft tumor [135]. Another important miRNA in regulating CSC function is miR-21. Many types of cancer, in addition to CSCs, have high expression of miR-21 [35, 47, 96]. It has been reported that miR-21 and hypoxia-inducing factor (HIF-1α) play an essential role in the regulation of breast CSCs’ proliferation [8]. An additional important miRNA in CSCs, tumor invasion, and metastasis is miR-26. It was found that miR-26 could inhibit the polycomb group protein, EZH2, a regulator of CSCs correlated with angiogenesis, invasion, and self-renewal capacity [7, 19]. In pancreatic cancer cells, re-expression of miR-26 was reported to downregulate the expression of EZH2, Oct4, Notch-1, and EpCAM that indicate its critical role in CSC modulation [9]. Another example of miRNA that is involved in CSC regulation is miR-34a, as it was mentioned to lower the expression of CSC genes such as CD44, CD133, and Notch-1 [55, 97] and suppress cancer proliferation [80]. Furthermore, miR-34 was found to act as tumor suppressor miRNA and correlated to the self-renewal characteristics of the CSCs by affecting apoptosis and self-renewal pathways [65]. It could inhibit Bcl2, Notch, and HMGA2, which eventually inhibit the self-renewal properties of CSCs and cancer progression [131]. Moreover, miR-200 family members play a significant role in cancer progression [60]. miR-200c downregulates the expressions of Bmil-1, Suz12, and Notch-1, regulators of CSC and EMT phenotypes and functions in numerous cancer cells [10, 74]. MiR-22 a, b, and c were detected in CSC-like (CD44 + /CD24 −) cells of breast cancer [111] and suppresses EMT in colorectal CSC [126]. Another important miRNA in CSC- elf regulation is miR-199b-5p. miR-199b-5p was found to inhibit the expression of the Notch pathway transcription factor; HES1that is correlated to CSC metastasis [45, 124]. The correlation between miRNAs and the hallmarks of cancer stem cells is represented in Fig. 5.

#### Correlation between LncRNAs and CSCs

**Fig. 6:**
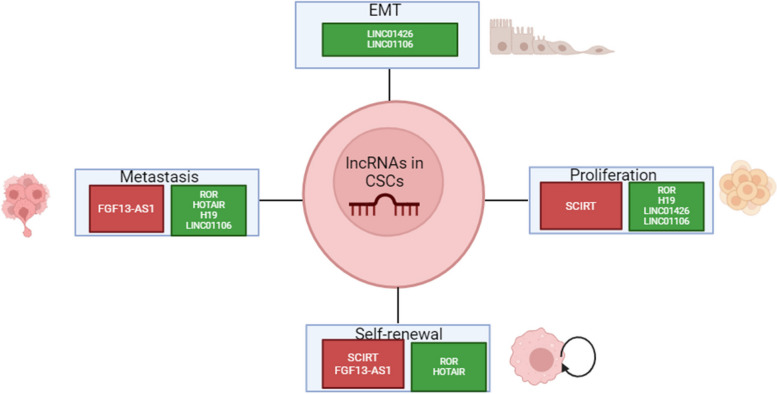
Correlation between lncRNA and the hallmarks of cancer stem cells. lncRNA that acts as promotor and suppressor are presented in green and red, respectively. EMT, epithelia mesenchymal transition

Many lncRNAs were found to regulate CSCs in various types of cancer throughout their different development steps (Xiwei Zheng, Cong Bi, Marissa Brooks 2015). The lncRNA ROR is upregulated in many types of cancers [44]. ROR targets the most important transcription factors for pluripotent stem cell phenotypes such as SOX2, OCT4, and NANOG [52]. Moreover, DNA and stem cell self-renewal could be impaired by ROR [44, 140]. ROR was found to act as regulator for gastric CSC differentiation and self-renewal. Additionally, the CD133-mediated invasion and proliferation of gastric CSCs may be associated with ROR [121]. Another important lncRNA is HOTAIR which is transcribed from the antisense strand of HOXC gene cluster located on chromosome 12 [113]. HOTAIR is used for cancer diagnosis, upregulated in different kinds of cancer, associated with metastasis, and enhances the self-renewal ability of CSCs, so regarded as a poor predictor of a patient’s prognosis for survival [155]. In a previous study, HOTAIR knockout was found to shrink the growth of tumor in vivo which makes targeting HOTAIR a promising therapy for cancer [78]. Considering lncRNA H19, another example of lncRNA, it is a maternally expressed gene and an estrogen-regulated transcript [71]. H19 represent a significant player in regulating cell differentiation, and its abnormal expression might end with enhancement of various cancers [16]. Moreover, H19 increased the tumorigenicity and liver metastasis in vivo, as well as the malignant behaviors of NSCs and the production of the stemness marker protein in vitro, which was mechanistically linked to the activation of the YAP/CDX2 signaling axis. Additionally, H19 stimulates the growth of colony in breast cancer cells and using siRNA to inhibit H19 reduces the colony-forming potential in RWPE-1 prostatic cells [12, 147]. LINC01426 was upregulated in lung adenocarcinoma and impacts Shh ubiquitination, triggering the activation of the Hedgehog pathway in lung CSCs. LINC01426 upregulation facilitates the proliferation and migration of cells, suppressing apoptosis, and affecting the expression of markers associated with EMT [20]. LINC01106 induced the proliferation, migration, and stem-like phenotype of colorectal cancer cells [49]. SCIRT (Stem Cell Inhibitory RNA Transcript) regulates the transcription of genes associated with self-renewal and cell cycle processes critical in the shift from breast CSCs to more actively dividing mature cancer cells. SCIRT was reported to reduce cell migration and its silencing results in the increased expression of genes related to the TGF and PI3K-Akt pathways. Additionally, SCIRT reduces transcription of genes linked to self-renewal activities, opposing the actions of SOX2 and EZH2 [138]. The lncRNA FGF13-AS1 suppresses the glycolytic activity and stem-like characteristics of breast cancer cells via a feedback loop involving FGF13-AS1, IGF2BPs, and Myc [90]. The correlation between LncRNAs and the hallmarks of cancer stem cells is represented in Fig. 6.

#### Correlation between SnoRNAs and CSCs

Growing experimental evidence indicates that small nuclear RNAs (snoRNAs) may possess functional roles that extend beyond their traditional involvement in ribosomal biogenesis, particularly in processes related to the cancer development [137]. Mutations and irregular expression patterns of snoRNAs have been documented in cancer, suggesting that snoRNAs could act as biomarkers or potential treatment targets for cancer [82]. SnoRNAs were significantly involved in the progression of CSCs. Zhou et al. discovered the importance of amino-terminal enhancer of split (AES), C/D box snoRNAs, in the promotion of self-renewal induced by the oncofusion gene AML1–ETO in leukemia cells [152]. C/D box snoRNAs exhibit heightened expression in leukemia and are closely associated with the frequency of leukemic stem cells in vivo. Moreover, DDX21, an RNA helicase that aids in rRNA modification, interacts with snoRNA/RNP complexes and leads to the reduction of AES levels, which diminishes the binding of DDX21 to the C/D box snoRNP complex containing FBL, NOP598, NOP56, and NCL, leading to the suppression of snoRNAs [152]. Knockdown of SNORD14D or SNORD35A disrupts the clonogenic potential of leukemia cells and delays the onset of leukemia [152]. Mannoor et al. illustrated that ALDH1 + served as a marker for CSCs. ALDH1 + cancer cells exhibit significant capabilities in self-renewal, proliferation, and tumorigenesis in vivo [92]. Twenty-two snoRNAs show distinct expression in ALDH + cancer cells. Pediatric high-grade gliomas (pHGGs) are aggressive malignant brain tumors originating from glial stem cells that showed a notable decrease in the expression of 36 snoRNAs from the HBII-52 snoRNA cluster. Additionally, mutations in h3f3a and TP53 resulted in substantial alterations in snoRNA expression in pHGG tissues [127, 151].

SNORA3 and SNORA43 were found to be upregulated in lung CSCs and exhibited an inverse correlation with the survival of non-small cell lung cancer (NSCLC) patients. SNORA42, along with CD133, another crucial marker for lung CSCs, was identified as particularly dysregulated in lung CSCs. Silencing SNORA42 suppressed the ability for self-renewal and tumor formation in vitro by promoting apoptosis and lowering the expression of stem cell-related genes such as OCT4, Nanog, Sox2, Notch1, Smo, and ABCS2. This indicates a potential link between SNORA42 and the regulation of key transcription factors associated with stem cells in lung cancer stem cells [92, 152]. Moreover, SNORD78 has been identified as upregulated in non-small cell lung CSCs and is essential for its self-renewal process [152].

SNORD89 exhibited high levels of expression in ovarian CSCs and was linked to unfavorable outcomes in ovarian cancer patients. Elevated SNORD89 levels resulted in heightened expression of stem cell markers, an increase in the proportion of cells in the S-phase of the cell cycle, as well as enhanced proliferation, invasion, and migration of ovarian cancer cells. Conversely, these effects were reversed upon the knockout of SNORD89. SNORD89 is suggested to function as an oncogene in ovarian tumors by enhancing cell stemness through the regulation of the Notch1-c-Myc pathway, consequently contributing to an unfavorable prognosis among ovarian cancer patients [156].

**Fig. 7:**
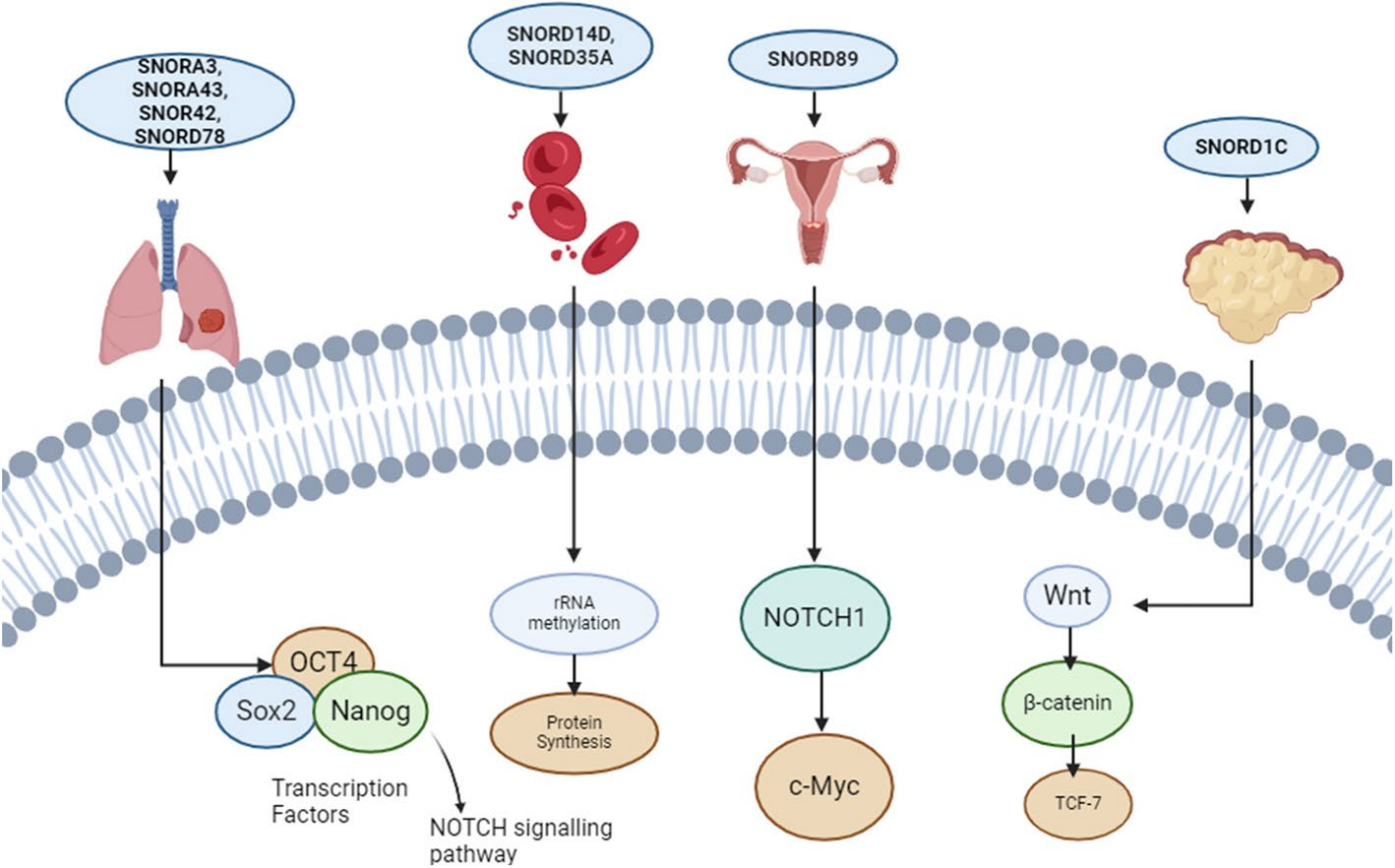
Correlation between snoRNAs, different types of cancer stem cells and their regulatory pathways

SNORD1C sustains stem cell characteristics and resistance to 5-FU by activating the Wnt signaling pathway in colorectal cancer. Depleting SNORD1C in colorectal cancer cell lines resulted in reduced cell proliferation and migration, along with an increase in apoptosis [87]. The correlation between snoRNAs, different types of CSCs, and their regulatory pathways are represented in Fig. 7.

#### Correlation between PiRNAs and CSCs

Small noncoding RNAs known as piwi-interacting RNAs (piRNAs) were first identified in Drosophila melanogaster in 2001 [3]. The four PIWI proteins that humans have are PIWIL1, PIWIL2, PIWIL3, and PIWIL4. These proteins are highly conserved across species. Conserved PAZ and MID domains found in PIWI proteins can identify the 3′ and 5′ ends of piRNA intermediates, respectively. Variations in the expression of piRNAs have been linked to the development and spread of several malignancies. Their mechanisms and roles correspond with several representative cancer hallmarks [100].

piR-823 influences the development of hematological malignancies, gastric cancer, colorectal cancer, and esophageal squamous cell carcinoma. piR-004800 protein either activates or inhibits the PI3K/AKT/mTOR pathway, and it is regulated by the sphingosine-1-phosphate receptor [91]. In multiple myeloma, downregulation of piR-004800 results in apoptosis and limits cellular proliferation. piR-Hep1 is upregulated in hepatocellular carcinoma and activates PI3K/AKT pathway, regulating proliferation, survival, and metabolism. Silencing piR-Hep1 suppresses cell viability and motility.

piRNA-PIWI axis is more active in undifferentiated cells than in differentiated ones. It maintains, multiplies, and survives CSCs because it controls epigenetic processes and is necessary for stem cell renewal. Thus, modifying this axis may offer a fresh approach to focusing on a subset of cancer cells that are essential to the growth of tumors, their resistance to therapy, and their recurrence. EMT induction increased the levels of piR-932, which interacted with PIWIL2, in CD44 + /CD24 − breast CSCs. Fibroblasts’ overexpression of PIWIL2 resulted in the acquisition of several characteristics of CSCs and an increase in the expression of numerous piRNAs, including the piRNA MW557525. Stem cell markers like CD24, CD133, KLF4, and SOX2 were not only suppressed by inhibitors of the piRNA MW557525 but also induced apoptosis [141].

piR-823 was elevated in breast CSCs. Increased proliferation and overexpression of the stemness genes POU5F1, SOX2, KLF4, NANOG, and h-TERT were brought about by piR-823 overexpression [36]. piR-2158 is downregulated in breast cancer, and it is downregulated significantly more in ALDH + breast CSCs than in ALDH − breast cancer cells. Reduced proliferation, migration, and invasiveness were seen with overexpression of piR-2158, along with a reduction in the expression of EMT markers. Surprisingly, after receiving piR-2158 mimetics, the CSC markers SOX2, OCT4, NANOG, and KLF4 were likewise downregulated [149].

Downregulation of PIWIL2 led to an increase in apoptosis and a decrease in the expression of markers for proliferation and apoptosis, such as STAT3/Bcl-XL/cyclin D1 [75]. The proliferation and invasion of cervical cancer cells were reduced when PIWIL2 was downregulated [43].

### Non-coding RNAs are promising therapeutics targets

Aberrant expression of non-coding RNAs is connected to many diseases, such as cancer. Additionally, evidence has revealed that cancer cells show distinct miRNA profiles compared to normal cells [61]. Many literatures identified and characterized the presence of self-renewal CSC subpopulation within the tumor [57]. Given that abnormal gene expressions are significant features in cancer, this might explain why epigenetic abnormalities relate to genetic alteration and resulting in CSC progression and stimulation [150]. These findings introduce non-coding RNAs as important promising targets for CSCs owing to their unusual expressions that correlated with CSC regulation. However, the development of miRNA/RNAi-based therapeutics needs many steps starting with miRNA profiling in cancer and normal cells, to the use of different RNAi-therapies in vitro and in vivo too. Moreover, many miRNAs have been characterized within the tumor as either oncogene or tumor suppressors, so there are two strategies to use noncoding RNA as cancer therapy. The first involves using miRNA mimics or miRNA expression vectors to restore the function of tumor suppressor ncRNAs, and the second entails blocking the activity of oncogenic ncRNAs using a variety of methods, including locked nucleic acids (LNA anti-miRs), tiny LNA anti-miRs, antagomirs, miRNA sponges, and siRNA [85]. An antagomiR, anti-miR-21 oligonucleotide, could be used to downregulate their expressions and decrease their ability to stimulate cancer by downregulating the expression of the oncogenic miRNA-21. Anti-miR-21 oligonucleotide decreased tumor growth in a xenograft mouse model by enhancing apoptosis and decreasing cell proliferation when transfected into breast cancer MCF-7 cells [72]. These results make antagomiRs a promising therapy for miRNA-related CSCs [112]. On the other hand, MiRNA mimics or lentiviruses should be utilized to restore these miRNAs’ capacity to prevent the formation of cancer, taking into account that tumor suppressor miRNAs drive tumor when under-expressed. For instance, miR-34a mimics were successful in blocking the G1 phase of the cell cycle, enhancing caspase-3 activation, and inhibiting bcl-2, Notch, and HMGA2 [41]. Also, the lentiviral miR-34a was observed to inhibit cancer cell expansion and tumorsphere formation in pancreatic cancer cells [95]. Other examples of miRNAs that act as tumor suppressors are miR-15a and miR-16–1. It was found that miR-15a and miR-16–1 expression was downregulated in the majority of leukemic cells, and the expression of Bcl-2 was upregulated [26]. Restoring miR-15a and miR-16–1 causes downregulation of Bcl-2 and activation of apoptosis in cancer cells. Additionally, suppression of these two miRNAs led to an upregulation of WNT3A, a protein belonging to the Wnt gene family that is essential for prostate cancer cell proliferation and tumor invasiveness [17]. As Bcl-2 and Wnt pathways are important for the self-renewal process in CSCs, miR-15a and miR-160–1 mimics could be used to target CSCs [33]. Although both strategies of function restoration or blockage could be applicable for miRNA because of their small size and location in cytosol, it is more difficult to apply both techniques for lncRNA. This occurs as lncRNAs, unlike miRNAs, could fold into secondary and advanced order structures, and how it works is challenging to predict its sequence information [119]. Also, lncRNA exists in the nucleus, making it more easily degraded than miRNAs, and the processes underlying lncRNAs are not well explained. However, blocking of lncRNA function is easily performed by many techniques, but the most common one is downregulation with RNA interference [84].

### Challenges beyond the use of noncoding RNA as cancer therapy

The key challenge that this approach presently faces is achieving the safe, efficient delivery of the oligonucleotides into the malignant tissue. The main reason is the attack of the unmodified oligonucleotide by the immune system making it unstable in the circulation. Conversely, alterations could potentially improve the affinity for targets and stability but necessitate additional delivery systems to achieve the desired biological outcomes. Various factors must be taken into account when selecting a delivery system, such as its resistance to serum nucleases, capability to evade the innate immune response, prevention of nonspecific interactions with serum proteins and unintended cells, avoidance of renal elimination, release from circulation to access target tissues, facilitation of cellular entry, and integration into RNA interference or other pertinent cellular mechanisms [68]. However, a recent study demonstrated that miRNAs can be enclosed within multivesicular bodies, which are subsequently released as exosomes into the extracellular environment. These exosomes have shown potential in targeting colorectal CSCs [46]. This is considered a natural delivery system but needs more understanding and investigation of the exosome function in different types of cancer. Another challenge that faces noncoding RNA as therapy is off targeting feature. Since miRNAs have the ability to target multiple mRNAs, the targets identified in model systems, whether cellular or animal, may not necessarily translate to clinical situations [37]. For lncRNAs, the challenges are greater because their mechanisms are not well-known, and they are more tissue-specific than protein-coding genes [38].

## Conclusion

Accumulating evidence suggested that cancer stem cells with self-renewal and differentiation abilities are significant players in cancer initiation, progression, and metastasis. Recent studies showed that noncoding RNAs regulated CSC progression by acting as cancer promoters or inhibitors. Here, we discuss this relation between CSCs and different types of noncoding RNAs. Additionally, we discuss the pros and cons of using noncoding RNAs as potential targeted therapy for cancer. However, this field of research needs more investigation to be well understood and yet many regulating mechanisms remain to be revealed.

## Data Availability

All data generated or analyzed during this study are included in this published article.

## Abbreviations

ASCs: Adult stem
CLL: Chronic lymphocytic leukemia
CSCs: Cancer stem cells
EMT: Epithelia mesenchymal transition
ESCs: Embryonic stem cells
HH: Hedgehog
iPS: Induced pluripotent stem
lncRNA: Long non-coding RNA
miRNA: MicroRNA
ncRNA: Non-coding RNA
NSCs: Normal stem cells
piRNAs: Piwi-associated RNAs
Pri-miRNAs: Primary hairpin miRNAs
RISC: RNA-induced silencing complex
snoRNAs: Small nucleolar RNAs
TRBP: Transactivation response RNA-binding protein
